# Supplementary material for: Convolutional networks for supervised mining of molecular patterns within cellular context
Source: Nat Methods. 2023 Jan 23;20(2):284–94. doi: 10.1038/s41592-022-01746-2 (PMC9911354; doi:10.1038/s41592-022-01746-2)
Supplement: Supplementary file 1 — Supplementary Notes 1 and 2, Figs. 1–12, and Tables 1–9. [file 41592_2022_1746_MOESM1_ESM.pdf]

nature methods

Article https://doi.org/10.1038/s41592-022-01746-2

Convolutional networks for supervised mining of molecular patterns within cellular context

In the format provided by the authors and unedited

Table of Contents

- **Supplementary Note 1**: Architectural description of 2D and 3D CNNs, post processing steps and evaluation metrics. Includes Supplementary Figure 1-4.
    - Supplementary Tables:
        - Supplementary Table 1: Selected hyperparameters of DeePiCt CNNs for different cellular features.
        - Supplementary Table 2: Construction of ground truth data for ribosomes
        - Supplementary Table 3: Construction of ground truth data for FAS
        - Supplementary Table 4: Organelle types in annotated tomograms
        - Supplementary Table 5: Specifications of 3D-CNN training and prediction rounds performed for ribosome ground truth construction
        - Supplementary Table 6: Specifications of 3D-CNN training and prediction rounds performed for FAS ground truth construction
- **Supplementary Note 2**: Considerations for structural analysis following CNN predictions under different image acquisition conditions. Includes Supplementary Figures 5-12 detailing subtomogram hierarchical classification results.
    - Supplementary Tables:
        - Supplementary Table 7: Cryo-EM data collection for VPP tomograms, refinement and statistics for ground truth subtomogram averages
        - Supplementary Table 8: Cryo-EM data collection for defocus tomograms, refinement and statistics for ground truth subtomogram averages
        - Supplementary Table 9: Cryo-EM data collection for defocus tomograms, refinement and statistics for subtomogram averages based on DeePiCt predictions

Supplementary Note 1

Architectural description of 2D and 3D CNN, post processing steps and evaluation metrics

The general architecture of the 3D and 2D CNN is based on the U-Net architecture[14] and consists of two consecutive symmetric paths: an encoder and a decoder path connected by skip-connections and a transitional base-layer (**Fig. 1a**), where:

(a) The encoder path is a sequence of alternated convolutional blocks and max-pooling layers. In turn, the convolutional blocks are a series of convolutions (kernel size $k = 3$, stride $s = 1$ and zero padding $p = 1$) followed by a rectified linear unit (ReLU). In the 3D CNN, optional batch normalization (BN) and dropout layers can be alternated (**Fig. 1a**). The max-pooling layer has fixed window size $w = 2$ and stride $s = 2$.

In our implementations, the initial convolutional block has a variable number of initial filters (IF) in its first convolutional layer, which must be set by the user. In all cases, subsequent convolutional blocks duplicate the number of feature maps from the previous level. The number of downsampling layers plus 1, is referred to as the network's depth (D), which is also a hyperparameter to be set by the user. It determines the size of the receptive field of the network. For the 2D CNN, the depth is a fixed value $D = 5$.

(b) The base of the CNNs is the transitional convolutional layer between the encoder and decoder paths.

(c) The decoder path is symmetric with respect to the encoder path, and is a sequence of alternated convolutional and upsampling (via transposed convolution) layers[70], with kernel size $k = 3$, stride $s = 1$ and padding $p = 1$. For the 3D CNN, analogous optional layers can be alternated between convolutional and upsampling blocks (BN and dropout). The encoder and decoder paths' associated parameters are denoted throughout the text as ED and DD, respectively.

Essential to the U-Net architecture are the *skip-connections*, which consist of concatenating the feature maps from the encoder to the decoder path at every level. The last activation layer is, in our case, a sigmoid function, compatible with the two available loss function choices, Dice and Generalized Dice[71,72]. For the latter we use the implementation of the artificial intelligence toolkit by MONAI[73]. By definition, this is a multi-label approach, where single voxels may belong to multiple semantic classes, and which frees the user from defining additional weighting parameters to compensate for class imbalance necessary for other loss function choices such as categorical cross-entropy. In our experience, the use of Dice (and Generalized Dice) Loss provided better qualitative results than cross-entropy, while in the multi-label network both Dice and Generalized Dice loss functions provide similar results (**Supplementary Fig. 1**).

The presence and the frequency of the structure in the training set is one of the parameters that in our experience largely determines whether the 3D CNN is able to learn. The user can set in the configuration file a minimum label presence ($0 \leq min\_label\_fraction \leq 1$), which indicates the proportion of labeled voxels in the patch that is required for it to be considered among the training patches. In this way, patches with too few labeled voxels are eliminated.

All models were trained using Adam Optimiser[74]. Application of these networks to unseen tomograms requires scaling of their pixel size to match (at least approximately) that of the training data.

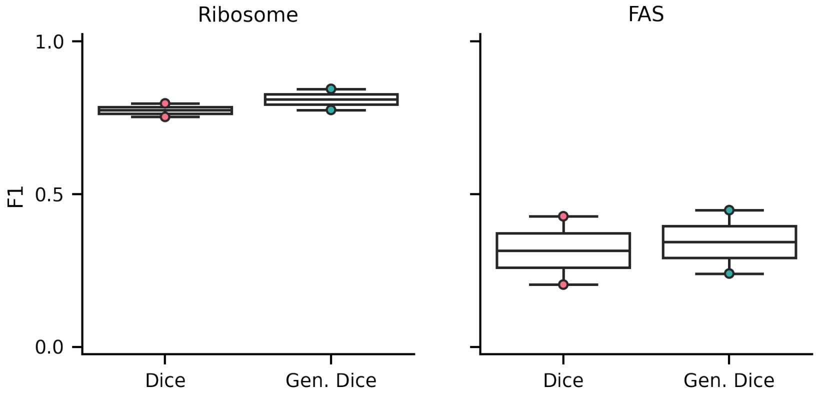

Supplementary Figure 1 | Comparison of Dice and Generalized Dice Loss functions for a multi-label network. The results of two multi-label networks, trained with Dice and Generalized Dice Loss functions, are evaluated. Both networks were trained for simultaneous segmentation of ribosomes and FAS, two highly unbalanced classes. The results show the F1 score on n=2 VPP tomograms, after being trained with 8 VPP tomograms. For the Dice Loss trained network, the results have a median F1 of 0.32 for FAS and 0.78 for ribosomes, while for the Generalized Dice Loss trained network, it has a median F1 of 0.34 for FAS and 0.81 for Ribosome. Boxplots' middle line marks the median and the edges indicate the 25th and 75th percentiles; whiskers encompass all data that are not considered outliers (calculated by the Seaborn boxplot function).

Data Augmentation

For the 2D CNN, tiles are randomly flipped and rotated in 90-degree increments during training to improve generalization.

Data augmentation for training the 3D CNN is available and optional, although it did not improve results in our hands (described below in relation to evaluation and training set size; **Supplementary Fig. 2**). The number of times that each of the original training examples is augmented is given by a parameter n_{DA} that can be set by the user. Each augmentation includes 4 different types of random volumetric transformations (applied to the original image and the labels, except in the case of noise), that we describe in what follows. Let A in $R^{n\times n\times n}$ be a single channel cubic image of side-length $n > 0$. Then:

1. **Additive Gaussian noise.** Given a predefined modulation factor $\epsilon > 0$, the Additive Gaussian noise transformation G_ϵ is defined by $G_\epsilon(A) = A + \epsilon B$, where $B_{ijk} \sim N(0,1)$ are independent and identically distributed random variables (i.i.d.r.v.), and $N(\mu, \sigma^2)$ denotes a normally distributed Gaussian random variable with mean μ and variance σ^2.
2. **Salt-and-pepper noise**. Given user-defined modulation factor $a > 0$ and a probability rate $1 > p > 0$, the salt-and-pepper noise transformation is defined by $S_{p,L}(A) = A + a * B$, where $B_{ijk} \sim U(p, [-1,1])$ are i.i.d.r.v., and $U(p, I)$ denotes a uniformly distributed random variable in a real interval I.
3. **Elastic transformation.** Given coordinate entries of A that are located at a *coarse* regular grid of thickness $n_c > 1$ are displaced by a random vector of i.i.d.r.v. uniform random variables. To achieve a smooth displacement vector field for all the points of the image A (i.e. on the *fine* grid), we generate the elastically transformed image as the interpolated vector field (via polynomial interpolation of order $k > 0$, defined by the user).

4. **Random rotation**. Given a probability of rotation $p \in (0,1)$ and angle range $\alpha \in (0,180)$, the image A is rotated with a probability p with and angle in $[-\alpha, \alpha]$, with respect to the z −axis.

Training set sizes for particle picking

The amount of data required for training a network is dependent on the selection of hyperparameters, which in turn define the number of trainable parameters in the network. Performance results of DeePiCt for ribosome and FAS (**Supplementary Fig. 2**) show that 300 particles are enough to achieve the maximum F1 score for both abundant and sparse particles. The hyperparameters considered in each case correspond to the optimal values stated in **Supplementary Table 1**.

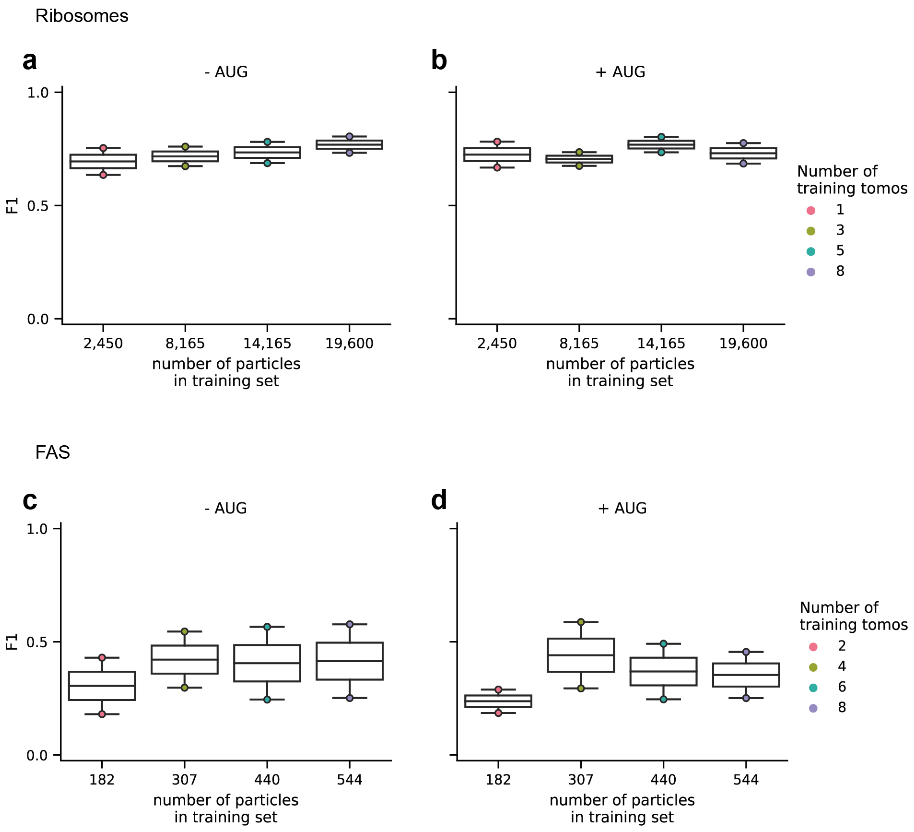

Supplementary Figure 2 | Performance with and without data augmentation across training set sizes. **a-d.** Plots show network prediction performance on n=2 tomograms over single independent experiments, each corresponding to variable training set sizes for ribosome (**a-b**) and FAS (**c-d**), without (- AUG, **a** and **c**) and with (+ AUG, **b** and **d**) data augmentation. For ribosomes (**a-b**), the naturally high numbers of particles per tomogram allow high F1 scores even when training on one tomogram (2,450 particles). FAS results (**c-d**) show that 300 particles are enough to get a fully trained network. In both cases data augmentation strategies including additive Gaussian noise, salt-and-pepper noise, and random rotations around the z-axis do not show clear advantages (**b**, **d**). Boxplots' middle line marks the median and the edges indicate the 25th and 75th percentiles; whiskers encompass all data that are not considered outliers (calculated by the Seaborn boxplot function).

3D CNN default hyperparameters

A default, starting point set of hyperparameters values for the 3D CNN for this study: $D = 2, IF = 4, BN = 0$, $ED = 0$, $DD = 0$, and $n_{DA} = 0$. We used them to later, through testing variations on them, study the optimal combinations of hyperparameters in the different segmentation and localization tasks (**Supplementary Table 1**).

2D CNN post-processing

Post-processing of the 2D CNN includes the reassembly of predicted tile segmentations, by cropping 48 px on each side to reduce artifacts around the edges. Areas of sets of tiles still overlapping are averaged after cropping. In order to remove inter-slide discontinuities, a Gaussian filter with parameter $\sigma > 0$ (default $\sigma = 5$) is then applied along the z axis.

3D CNN post-processing

1. Thresholding of the final probability map outputted by the last layer of the neural network (default threshold is the unbiased value 0.5, but can be set by the user in the configuration file).
2. Clustering of the resulting thresholded map via the *connected component labeling label* algorithm, using the function *morphology.label* of the *scikit-image* python library[75].
3. Integration with other segmentation maps, *e.g.* with the output of the 2D CNN. Importantly, a subsequent selection of clusters is made with respect to a *region_mask* provided by the user, which consists of an auxiliary binary image defining a region of interest (**Fig 1b**). We then allow 3 types of options (*intersection*, *contact*, or *colocalization*) for employing the *region_mask* through the corresponding *contact_mode* parameter, as follows (**Supplementary Fig. 3)**:
    a. *contact_mode: intersection:*
    The region_mask is used to directly mask the output of point 2, by considering only the overlapping region, *e.g.* including particles that are within an organelle's mask.
    b. *contact_mode: contact*
    Only clusters that have voxels in common with the region of interest are kept, *e.g.* selecting NPC on the nuclear envelope.
    c. *contact_mode: colocalization*
    Only clusters whose centroid is located at a given distance (*colocalization_radius*) from the region of interest are kept, *e.g.* particles within a distance to an organelle.

    At this step, only clusters within the size range $[m, M]$ are kept, where $0 \leq m < M \leq \infty$ are defined by the user in the configuration file.
4. If the *calculate_motl* parameter is set to *True*, a list of the clusters' centroids resulting from previous steps is saved as a 4-columns comma separated values (csv) file, where the first column is the score (cluster size), and the three remaining columns indicate the (x, y, z) centroid voxel coordinates. The list is sorted according to decreasing-score.

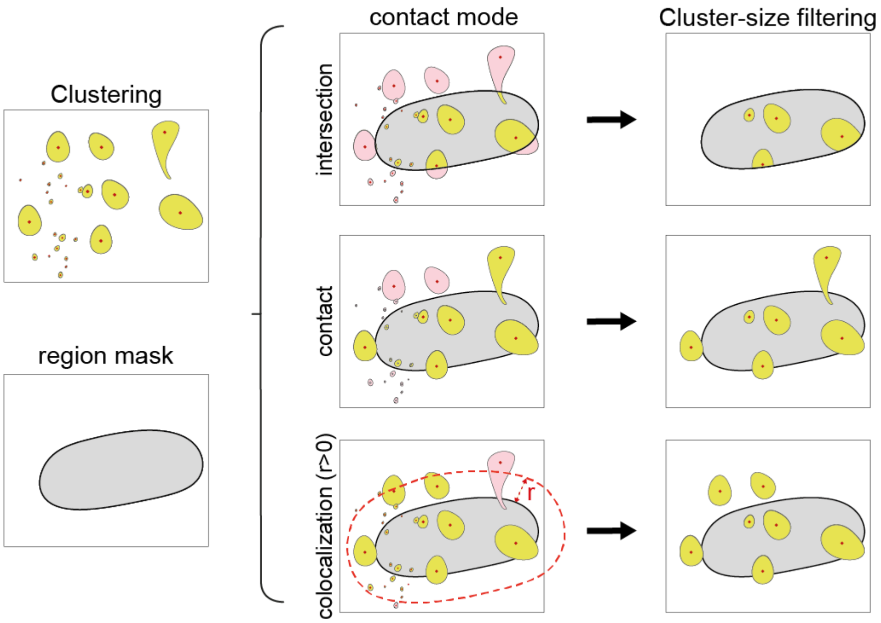

Supplementary Figure 3 | 3D CNN post-processing steps. The clustered 3D CNN output and a *region mask* (left) are combined according to the three contact modes (middle), where parts of the predicted clusters are kept (yellow), and others discarded (pink). The resulting clusters are filtered by number of voxels, according to minimum and maximum bounds set by the user (right). Dots inside clusters represent corresponding centroids.

Segmentation evaluation

A 3D cellular segmentation probabilistic prediction (resulting either from the 2D or the 3D CNN) is a map whose value at every voxel (i, j, k) is the probability $0 \leq p_{i,j,k} \leq 1$of belonging to the structure of interest (**Supplementary Fig. 4a**). Setting a threshold $0 \leq t \leq 1$ induces a binarized prediction (such that if $t \leq p_{i,j,k}$ the voxel value is set to 1 and the rest to 0; **Supplementary Fig. 4b**), whose quality can be evaluated by comparison to a ground truth binary image, according to different metrics:

1. Voxel-based precision (P) is defined by:

$$P = \frac{vol(tp)}{vol(tp)+vol(fp)},$$

   where vol indicate the volume (number of entries different from 0). Thus, P is the proportion of voxels in the predicted segmentation that *truly* belong to the wanted structure (**Supplementary Fig. 4c**).

2. Voxel-based recall (R) is defined by:

$$R = \frac{vol(tp)}{vol(tp)+vol(fn)},$$

   thus indicating the proportion of voxels of the ground truth segmentation that were correctly recovered by the prediction (**Supplementary Fig. 4c**).

3. Voxel-based F1-score (also known as Sørensen-Dice coefficient, **Supplementary Fig. 4c**): A common metric to calculate the performance of the method by combining the P and R values simply through the voxel-F1 score, defined by their harmonic mean:

$$voxel - F_1 = \frac{2*P*R}{P+R}.$$

Notice that here no account of the dependence with respect to the t (threshold) value is used.

4. Area under the precision-recall curve (AUPRC, **Supplementary Fig. 4e**): Since the prediction is dependent on the threshold value $0 \leq t \leq 1$ applied to the probability map, both precision and recall depend on it as well, $P = P(t)$ and $R = R(t)$. The area under the precision-recall curve (AUPRC) is the area under the parametrized curve $(P(t), R(t))$, which defines a metric useful to summarize the power of the method across the threshold (and probability) dimension.

Particle detection evaluation

In the case of particles as discrete objects that are small enough to be defined by their coordinates, the evaluation of the object detection task requires the following definitions (**Supplementary Fig. 4d**):

1. Given a sorted list of predicted particles' centroids, one by one (in that order) they are either classified as false positives (fp) or true positives (tp).
2. A predicted particle coordinate is considered a true positive if and only if given a pre-defined tolerance radius r ($r = 10$ vox for both ribosome and FAS), there exists a ground-truth coordinate not previously matched to any other predicted particle.
3. Then the coordinate-based precision (p), recall (r), F1 score and area under the precision-recall curve (AUPRC) are defined by:
$p = \frac{tp}{tp+fp}$, $r = \frac{tp}{tp+fn}$, $F_1 = \frac{2*p*r}{p+r}$, and AUPRC is the area under the curve defined by the (p, r) points when parametrized with respect to the cluster number (from 0 to all).

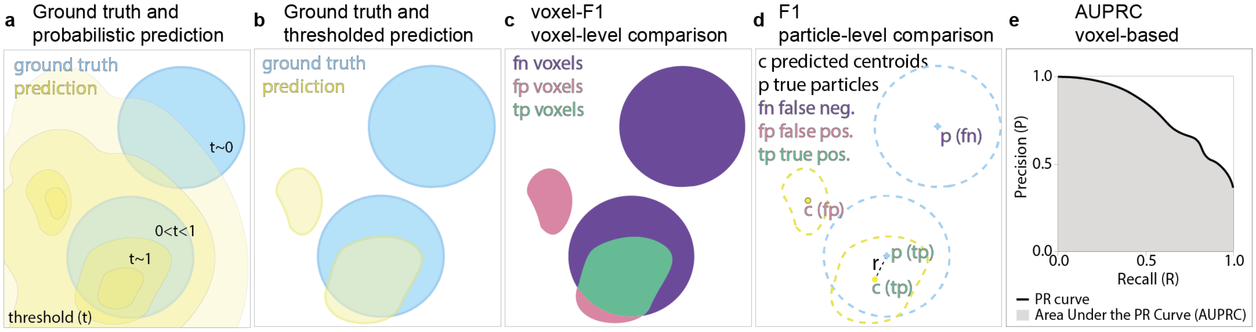

Supplementary Figure 4 | Performance measures. **a**. A binary ground truth (blue) voxel representation and a probabilistic prediction (yellow) of a structure of interest are provided. **b**. For any given threshold $0 \leq t \leq 1$, the prediction can be binarized (yellow) and compared to the ground truth (blue). **c**. The voxel-F1 score is the harmonic mean of voxel-based precision (P) and recall (R), computed according to the formulas in the text, considering true positive voxels as the voxels that are in the overlap (green), false positives are voxels that belong to the prediction but not to the ground truth (pink), and false negatives are voxels that are in the ground truth but not in the prediction (purple). **d**. Particle-based comparisons, on the contrary, treat each cluster of the prediction and ground truth as a separate *particle*, and thus centroids (rather than voxels) are compared. Given a radius r preset by the user, a predicted centroid (c) is a true positive (tp) when it lays within r of a true centroid (p). False positives (fp) are predicted centroids located beyond r from any true centroid, and false negatives (fn) are true centroids that are located beyond r from all predicted centroids. According to this convention, one can calculate the particle-based precision (p) and recall (r) as described in the text. **e**. The voxel-based Precision-Recall pairs $(P(t), R(t))$ parametrized by the threshold t, define the Precision-Recall Curve, whose area under the curve (AUPRC, in gray), defines a performance metric that accounts for the quality of the method across the prediction score.

Merging several lists for ground truth construction

To account for possible duplicates when merging lists of particle coordinates from different methods (e.g. manually-, TM- and CNN-originated lists), we integrate them by restricting the distance between centroid coordinates, *i.e.* with the constraint $d(p,q) > 1$, where the elliptic distance $d(p,q)$ is defined by:

$$d(p,q)^2 = \frac{1}{a^2}(p_1 - q_1)^2 + \frac{1}{b^2}(p_2 - q_2)^2 + \frac{1}{c^2}(p_3 - q_3)^2,$$

for $(a,b,c) = (9,9,15)$, and where $p = (p_1, p_2, p_3)$ and $q = (q_1, q_2, q_3)$.
The choice of the elliptic distance coefficients takes into account both the effect of elongation along the z-axis in the cryo-ET volume and the size of the particle in voxels of the tomogram (1 vox = 13.48 Å).

Supplementary Table 1: Selected hyperparameters of DeePiCt for varying cellular structures. Performances were calculated by cross-validation (CV) within the same domain, or in the case of microtubules and actin networks, in evaluations for generalization across cellular species with respect to expert annotations in the HeLa cell tomogram[+]. DeePiCt networks were applied to the test datasets with the following *region mask* and *contact mode* combinations: cytosol and intersection for ribosome and FAS; cytosol and contact for membranes; nuclear envelope and contact for NPCs; cytosolic volume and intersection for microtubules and actin filaments.

Cellular feature	Method	Depth (D)	Initial filters (IF)	BN	Median Performance	Training dataset	Testing dataset
Cytoplasm	2D CNN	5	16	1	AUPRC=0.97	VPP	VPP
Organelles	2D CNN	5	16	1	AUPRC=0.92	VPP	VPP
Ribosomes	DeePiCt	2	4	1	F1=0.79	VPP	VPP
FAS	DeePiCt	2	16	1	F1=0.46	VPP	VPP
Membranes	DeePiCt	2	4	1	voxel-F1=0.79	VPP	VPP
NPCs	DeePiCt	3	16	1	voxel-F1=0.24	defocus*	defocus*
					voxel-F1=0.47	defocus*	defocus* (high quality)
					voxel-F1=0.19	defocus*	defocus* (lower quality)
Microtubules	DeePiCt	2	4	1	voxel-F1=0.83[+]	VPP	HeLa cell
Actin	DeePiCt	3	8	0	voxel-F1=0.10[+]	VPP	HeLa cell

Supplementary Table 2 | Construction of ground truth data for ribosomes.
* with elliptical constraint (a,b,c)= (9,9,15).

		Step 1						Step 2			Step 3		Integration
		Step 1A			Step 1B		Step 1						
VPP	Tomogram	Method	No. of peaks before visual inspection	No. of peaks after visual inspection	Method	No. of particles	Total no. of particles in initial annotation	Method	No. of new particles predicted	No. of new particles after visual inspection	Method	No. of particles	Total ground truth after integration
TRUE	TS_0001	TM	2000	1371	manual	343	1714	3 rounds DeePiCt	910	798	manual	394	2450
TRUE	TS_0002	TM	2000	1662	manual	121	1783	3 rounds DeePiCt	1211	840	manual	415	2342
TRUE	TS_0003	TM	2000	1260	manual	290	1550	3 rounds DeePiCt	1351	821	manual	481	2429
TRUE	TS_0004	TM	2000	1262	manual	369	1631	3 rounds DeePiCt	1868	1078	manual	681	2967
TRUE	TS_0005	TM	2000	1391	manual	888	2279	3 rounds DeePiCt	1924	1181	manual	517	3571
TRUE	TS_0006	TM	2000	981	manual	340	1321	3 rounds DeePiCt	530	182	manual	238	1336
TRUE	TS_0007	TM	2000	495	manual	117	612	3 rounds DeePiCt	330	179	manual	131	617
TRUE	TS_0008	TM	2000	1066	manual	796	1862	3 rounds DeePiCt	788	447	manual	668	2744
TRUE	TS_0009	TM	2000	1243	manual	2216	3459	3 rounds DeePiCt	391	197	manual	421	3482
TRUE	TS_0010	TM	2000	1377	manual	1447	2824	3 rounds DeePiCt	605	476	manual	563	3373

FALSE	TS_0011	TM	3000	533	manual	87	620	3 rounds DeePiCt	464	261	manual	621	838
FALSE	TS_0012	TM	3000	881	manual	-	881	3 rounds DeePiCt	711	274	manual	1037	1673
FALSE	TS_0013	TM	3000	718	manual	-	718	3 rounds DeePiCt	2189	882	manual	5268	5305
FALSE	TS_0014	TM	3000	713	manual	-	713	3 rounds DeePiCt	1227	611	manual	2174	2897
FALSE	TS_0015	TM	3000	682	manual	-	682	3 rounds DeePiCt	915	388	manual	2245	2783
FALSE	TS_0016	TM	3000	770	manual	-	770	3 rounds DeePiCt	1160	575	manual	3013	3783
FALSE	TS_0017	TM	3000	590	manual	-	590	3 rounds DeePiCt	1118	448	manual	1412	1646
FALSE	TS_0018	TM	3000	537	manual	-	537	3 rounds DeePiCt	2081	1014	manual	1906	2813
FALSE	TS_0019	TM	3000	548	manual	-	548	3 rounds DeePiCt	1052	373	manual	1742	1815
FALSE	TS_0020	TM	2000	358	manual	-	358	3 rounds DeePiCt	1009	364	manual	2239	2348

Supplementary Table 3 | Construction of ground truth data for FAS.
* with elliptical constraint (a,b,c)= (9,9,15).

		**Step 1**		**Step 2**				**Step 3**			
VPP	Tomogram	Method	No. of particles	Method	No. of peaks	No. of true positive peaks recovered from step 1	No. of new true positive peaks after visual inspection	Method	No. of particles	Total	Total after integration*
TRUE	TS_0001	manual	86	1 round DeePiCt	171	75	14	manual	2	102	101
TRUE	TS_0002	manual	50	1 round DeePiCt	84	47	15	manual	16	81	81
TRUE	TS_0003	manual	45	1 round DeePiCt	70	38	8	manual	6	59	59
TRUE	TS_0004	manual	84	1 round DeePiCt	134	73	18	manual	10	112	112
TRUE	TS_0005	manual	27	1 round DeePiCt	71	27	20	manual	11	58	58
TRUE	TS_0006	manual	69	1 round DeePiCt	47	40	1	manual	5	75	75
TRUE	TS_0007	manual	25	1 round DeePiCt	27	16	2	manual	13	40	40
TRUE	TS_0008	manual	46	1 round DeePiCt	106	37	14	manual	16	76	75
TRUE	TS_0009	manual	-	1 round DeePiCt	54	-	34	manual	30	64	64
TRUE	TS_0010	manual	-	1 round DeePiCt	166	-	34	manual	32	66	66
FALSE	TS_0011	manual	9	1 round DeePiCt	13	11	2	manual	5	16	16
FALSE	TS_0012	manual	27	1 round DeePiCt	93	46	22	manual	7	56	56
FALSE	TS_0013	manual	29	1 round DeePiCt	251	60	41	manual	6	76	76
FALSE	TS_0014	manual	27	1 round DeePiCt	89	47	12	manual	3	42	42
FALSE	TS_0015	manual	38	1 round DeePiCt	116	63	17	manual	9	64	64
FALSE	TS_0016	manual	19	1 round DeePiCt	127	37	11	manual	7	37	37
FALSE	TS_0017	manual	7	1 round DeePiCt	81	15	10	manual	5	22	22
FALSE	TS_0018	manual	8	1 round DeePiCt	243	19	9	manual	1	18	18
FALSE	TS_0019	manual	9	1 round DeePiCt	64	20	3	manual	5	17	17

FALSE	TS_0020	manual	5	1 round DeePiCt	195	7	8	manual	5	18	18

Supplementary Table 4 | Organelle types in annotated tomograms.
0 = not present, 1 = present, 1*= present in a small volume fraction, NI = not identified, ribosome-excluded region in the cytosol.

Dataset	Tomogram	cytoplasm	mitochondria	vesicle	tube	ER	nuclear envelope	nucleus	vacuole	lipid droplet	Golgi	NI
VPP	TS_0001	1	1	1	1	1	0	0	0	0	0	0
VPP	TS_0002	1	0	1	0	1	1	1	1	0	0	0
VPP	TS_0003	1	1	1	0	1	0	0	1	0	0	0
VPP	TS_0004	1	1	1	0	1	0	0	1	0	0	0
VPP	TS_0005	1	0	1	1	1	0	0	0	0	0	0
VPP	TS_0006	1	1*	1	0	1	1	1	0	0	1	0
VPP	TS_0007	1	1	0	0	1	1	1	0	0	0	0
VPP	TS_0008	1	1	1	0	1	0	0	0	0	1	1
VPP	TS_0009	1	0	1	0	1	1	1	1	0	0	0
VPP	TS_0010	1	0	1	0	1	1	1	0	0	0	0
defocus	TS_0011	1	0	1	0	1	0	0	1	0	0	0
defocus	TS_0012	1	0	1	0	1	1	1	1	0	1	0
defocus	TS_0013	1	0	1	0	0	0	0	1	1	1	0
defocus	TS_0014	1	1	1	0	1	0	0	1	0	0	0
defocus	TS_0015	1	0	1	0	1	0	0	0	0	1	0
defocus	TS_0016	1	1	1	0	1	0	0	1	0	1	0
defocus	TS_0017	1	0	1	0	0	1	1	1	0	0	0
defocus	TS_0018	1	0	1	1	1	0	0	1	1	0	0
defocus	TS_0019	1	0	1	0	0	1	1	0	0	0	0
defocus	TS_0020	1	1	1	0	1	1	1	0	0	1	0

Supplementary Table 5: Specifications of three 3D-CNN training and prediction rounds performed for ribosome ground truth construction. Each of the rounds in step 2 of the ribosome ground truth construction consisted of training 3 different 3D CNNs with default parameters except the IF number (**Supplementary Note 1**). The first training was performed on manually curated template matching (TM) results of the corresponding dataset (VPP or defocus). For VPP, in the second round, the 3D CNN were trained on aggregated TM results and first round predictions; whereas the third CNN round was trained on all previous aggregated results. For defocus, the second and third round of 3D CNNs corresponded to the VPP 3D CNNs of first and second rounds, respectively.

training round	Training dataset	Prediction dataset	Training set annotations	Initial filters	output
1	VPP	VPP	TM annotations	IF=4, 8, and 32	vpp_output_round1
2	VPP	VPP	TM annotations + vpp_output_round1	IF=4, 8, and 32	vpp_output_round2
3	VPP	VPP	TM annotations + vpp_output_round1 + vpp_output_round2	IF=4, 8, and 32	vpp_output_round3
1	defocus	defocus	TM annotations	IF=4, 8, and 32	def_output_round1
2	defocus	defocus	TM annotations + def_output_round1	IF=4, 8, and 32	def_output_round2
3	VPP	defocus	TM annotations + vpp_output_round1 + vpp_output_round2	IF=4, 8, and 32	def_output_round3

Supplementary Table 6: Specifications of 3D-CNN training and prediction rounds performed for FAS ground truth construction. Step 2 in FAS ground truth construction consisted of a single round of 3D CNN training, performed separately per dataset type (VPP and defocus). The round consisted of 3 networks with default parameters (**Supplementary Note 1**) except for the IF number, set to IF=4, 8, and 32. The networks were trained on an initial incomplete manual picking.

Training dataset	Prediction dataset	Training set annotations	Initial filters	output
VPP	VPP	Initial manual annotations	IF=4, 8, and 32	fas_vpp_output_round1
defocus	defocus	Initial manual annotations	IF=4, 8, and 32	fas_def_output_round1

Supplementary Note 2

Considerations for structural analysis following CNN predictions under different imaging conditions

Cryo-ET datasets of *S. pombe* utilized in this study were derived from cryo-FIB lamellae of wild-type, native, yeast cells. 20 tomograms used for comprehensive annotations were acquired with similar parameters in a dose-symmetric tilt scheme[58] (**Supplementary Tables 7-9, Methods**), with the only difference being imaging with conventional defocus-only (defocus) or the usage of a VPP in addition. All ribosome and FAS particles annotated in the two datasets (defocus and VPP) were subjected to subtomogram analysis, including CTF correction utilizing CTF fitting and 3D CTF model creation in Warp, and 3D refinements, hierarchical, and focused 3D classifications in RELION. During this structural analysis we made several observations, which are discussed in the following.

As described previously[30,59,76], acquiring tomography data with a VPP emphasizes low frequencies. Pronounced low frequencies lead to improved image contrast, which can facilitate data mining. With our method, more particles were detected in VPP than in defocus ground truth annotations of FAS complexes, which represent more challenging targets for data mining due to their low abundance and hollow structural signature. The increased SNR in VPP was also advantageous in hierarchical classifications of ribosomes (**Supplementary Fig. 5**), while no difference was apparent for FAS likely due to the low particle numbers (**Supplementary Fig. 6**). For VPP, all ribosomes clustered in defined averages which confirmed the high performance of DeePiCt and enabled the classification of a subset of 60S large subunits (**Supplementary Fig. 5**). In comparison, most defocus ribosomes from ground truth annotations or DeePiCt predictions clustered in poorly defined classes and no 60S large subunits could be separated even when classification results were improved by optimized particle poses (coordinates and orientations) after multi-particle refinement in M[22] **(Supplementary Fig. 7, 8**). In addition, in classifications focused on an area close to the head of the ribosomal small subunit and at the peptide exit tunnel, the additional head density was already apparent in 2D slices of the respective classes in the VPP data (**Supplementary Fig. 9 and 10**, respectively). Similarly, ribosomes close to ER and mitochondria that clustered into classes with adjacent membrane densities are more pronounced in VPP (**Supplementary Fig. 11 and 12**, respectively).

However, fine structural details that provide insights into functional conformations of molecular complexes are lost or cannot be recovered in the final reconstructions of VPP tomography data[30]. This has been suggested to be caused by inaccurate weighing[30] and a signal loss at high spatial frequencies[77]. The preservation of high-resolution information in defocus in comparison to VPP becomes especially apparent when looking at fine structural details in the subtomogram averages. Densities fitting ACPs of FAS (**Extended data Fig. 8 d-e**), P-site tRNAs (**Extended data Fig. 8 j-k**) inside the 80S ribosome and densities connecting ribosomes to an adjacent mitochondrion membrane (**Supplementary Fig. 12**) could only be resolved in defocus data.

We conclude that the usage of a VPP in tomography acquisition facilitated data mining, including the demonstrated domain generalization of applying DeePiCt models trained in VPP to defocus, and improved 3D classifications. This makes it a valuable tool that ultimately provides benefits at the exploratory phase of structural analysis, helping the design of experiments and analysis pipelines that can eventually be performed on defocus data from which higher resolution reconstructions can be obtained.

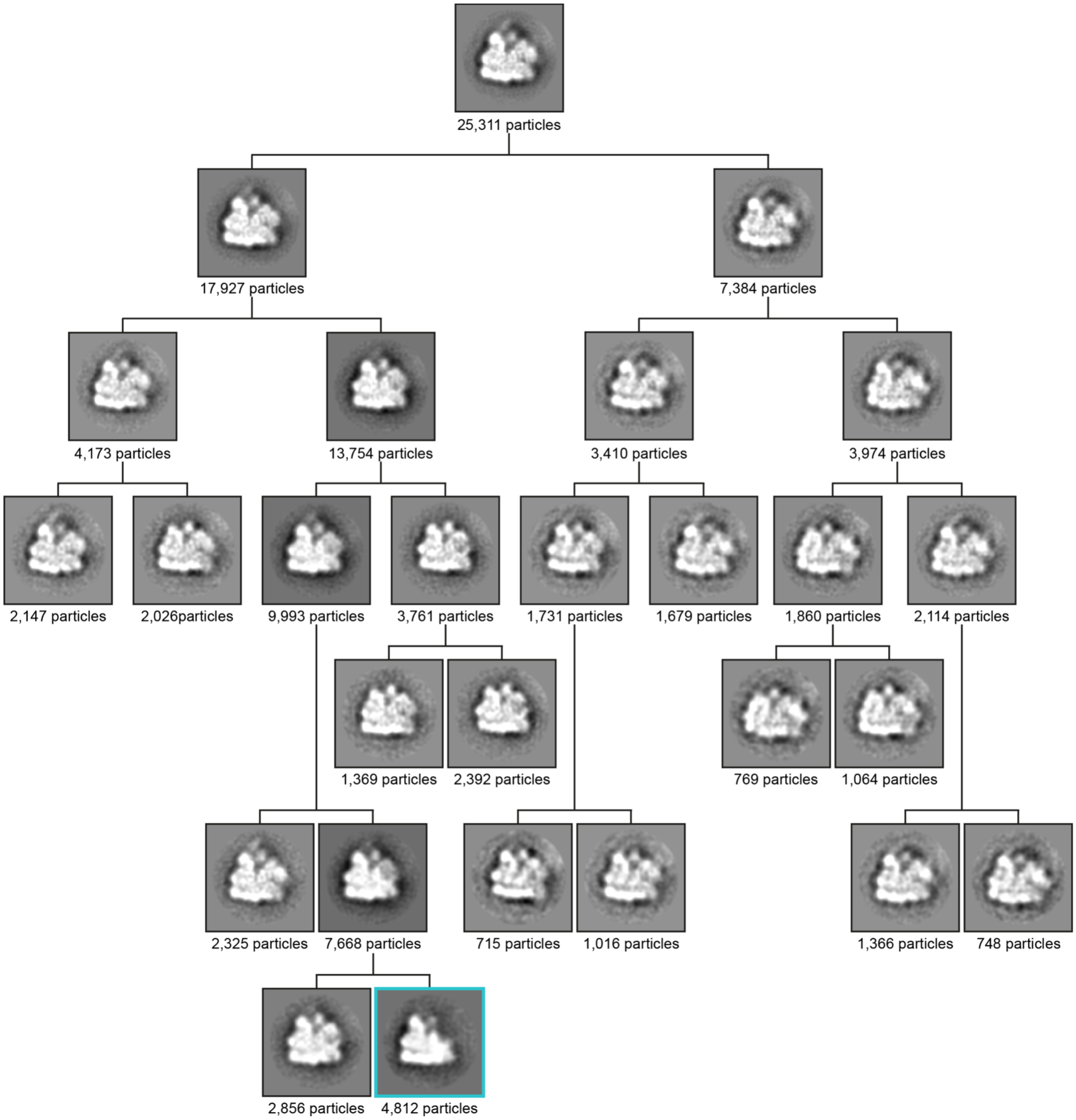

Supplementary Figure 5 | Hierarchical 3D classification of cytosolic ribosomes in VPP ground truth. 2D slices through 3D class averages of iteration 25 are displayed. The highlighted class (cyan box) shows the 60S large ribosomal subunit class.

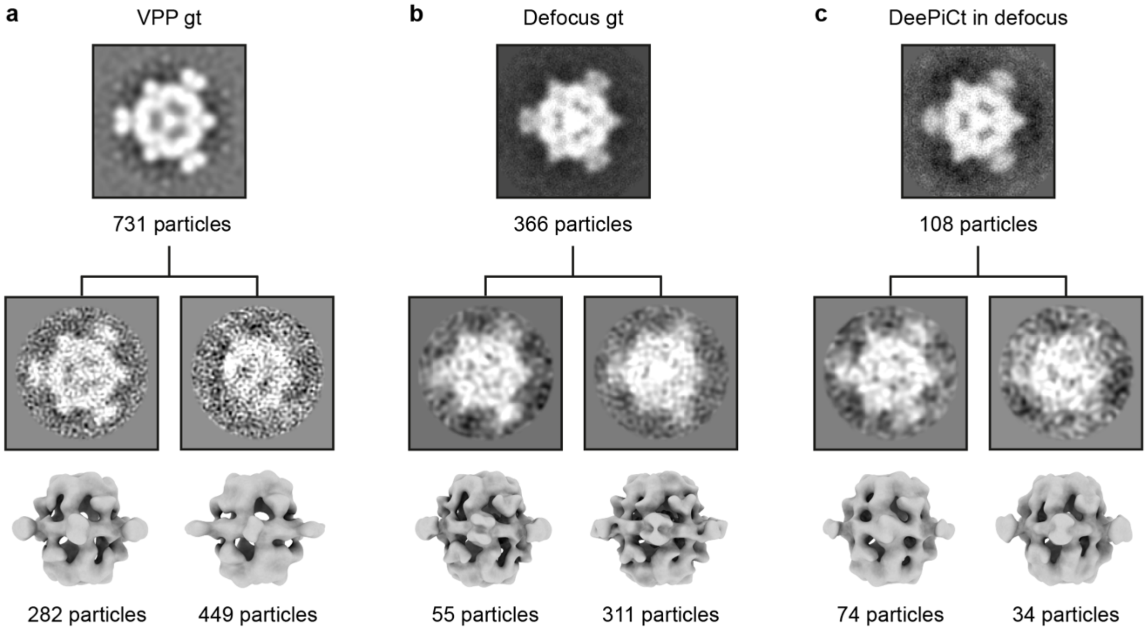

Supplementary Figure 6 | 3D classification of VPP and defocus ground truth, and DeePiCt prediction in defocus dataset for FAS. a-c. 2D slices through 3D-refined subtomogram average of FAS using D3 symmetry recovered from VPP ground truth (gt), defocus gt or DeePiCt prediction on defocus datasets. 3D classifications separated in all 3 datasets into two classes of which one is better structurally defined than the other. 3D refinements for defocus were performed in M.

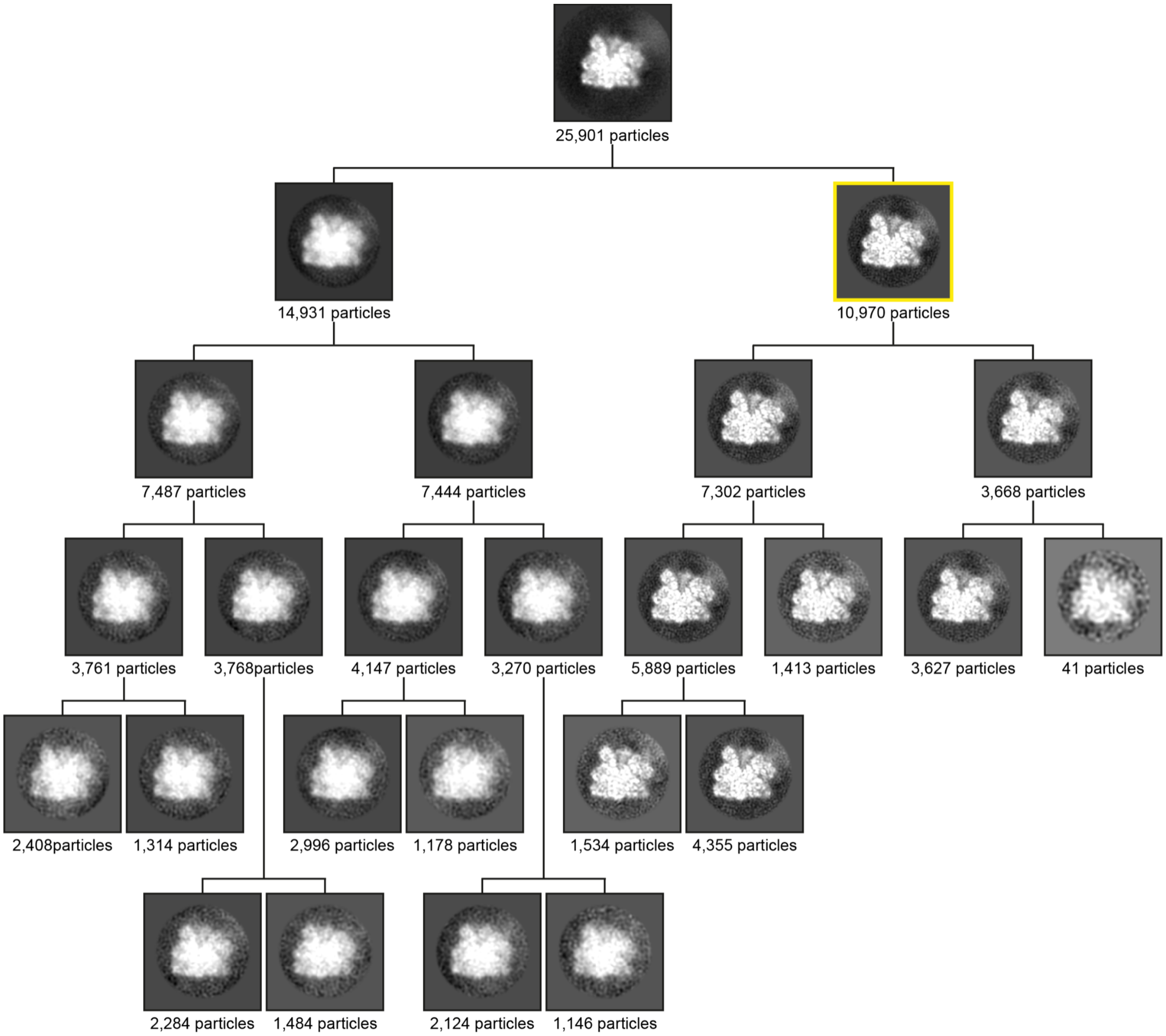

Supplementary Figure 7 | Hierarchical 3D classification of cytosolic ribosomes in defocus ground truth starting from M-refined alignments. 2D slices through 3D class averages of iteration 25 are displayed. The highlighted class (yellow box) was further refined in M, resulting in a well aligned class with a resolution of 9.3 Å.

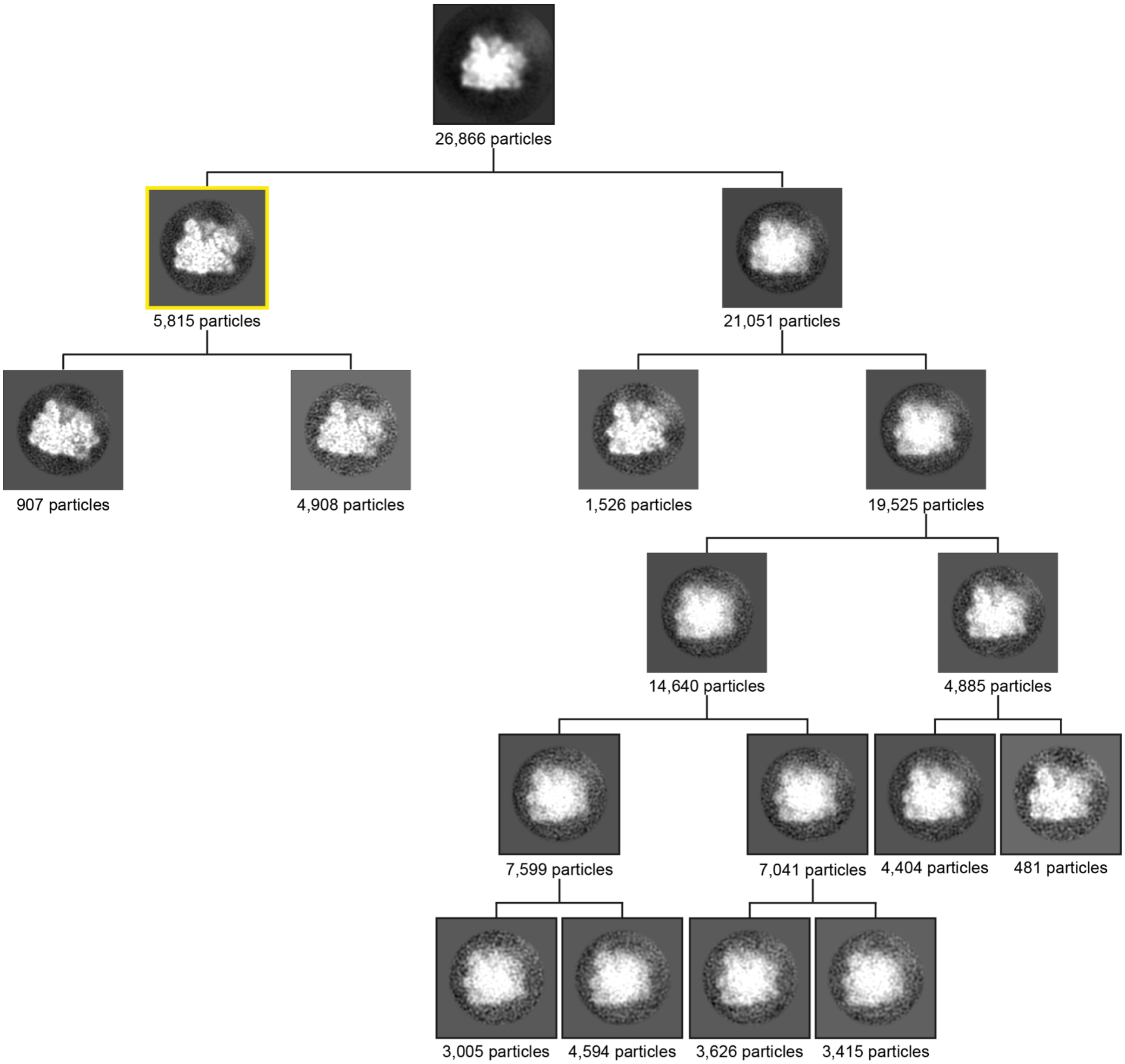

Supplementary Figure 8 | Hierarchical 3D classification of cytosolic ribosomes from DeePiCt predictions in defocus data starting from M-refined alignments. 2D slices through 3D class averages of iteration 25 are displayed. The highlighted class (yellow box) was further refined in M resulting in a well-aligned class with a resolution of 9.4 Å. The right side of the classification representing the vast majority of ribosomes does not converge into a well-defined map.

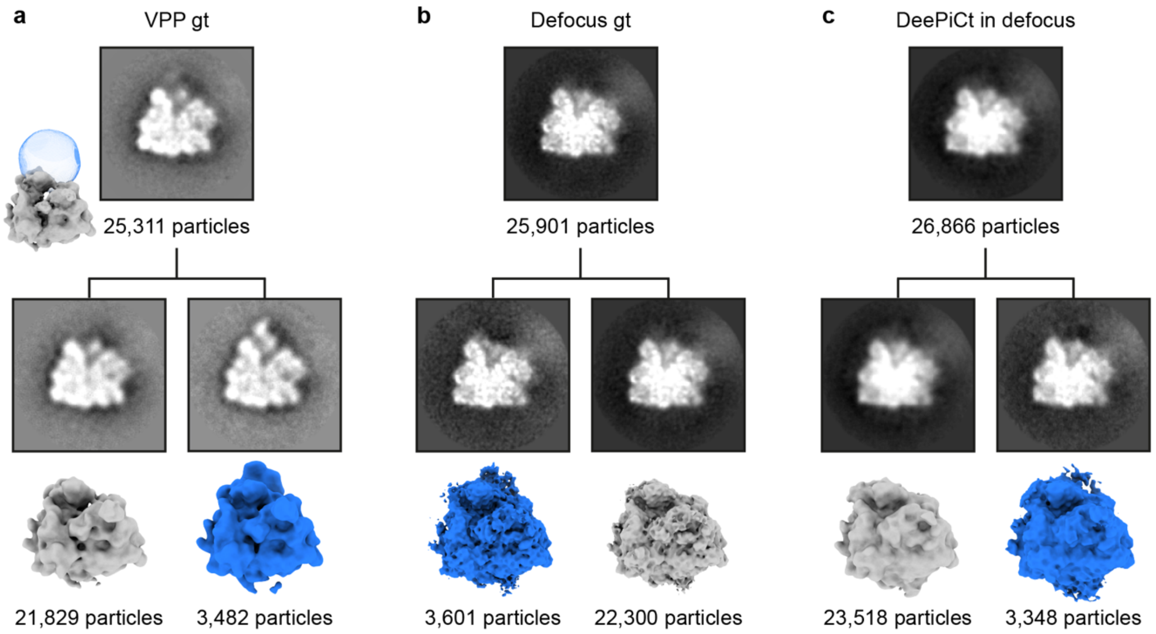

Supplementary Figure 9 | 3D classifications and refinements of cryo-EM densities in proximity to the head of the 40S small ribosomal subunit in VPP and defocus ground truth, and DeePiCt predictions in defocus datasets. a-c. 2D slices through 3D-refined subtomogram average of all cytosolic ribosomes from VPP ground truth (gt), defocus gt or DeePiCt predictions on defocus datasets. Focused 3D classifications (blue sphere mask displayed in a) separated in all 3 datasets one class (dark blue) with an additional density close to the head of the 40S small ribosomal subunit. 3D refinements for defocus were performed in M.

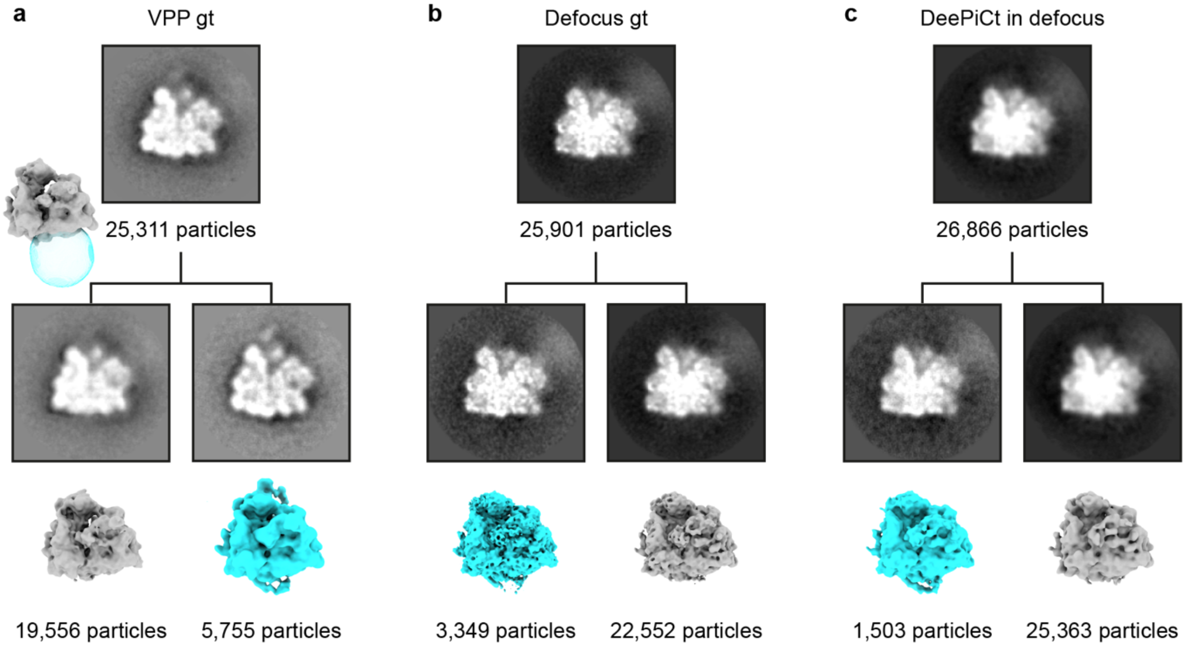

Supplementary Figure 10 | 3D classifications and refinements of ribosomes at the exit tunnel in VPP and defocus ground truth, and DeePiCt predictions in defocus datasets. a-c. 2D slices through 3D-refined subtomogram average of all ribosomes from VPP ground truth (gt), defocus gt or DeePiCt prediction on defocus datasets. Focused 3D classifications (cyan sphere mask displayed in a) separated in all 3 datasets one class (cyan) with an additional exit tunnel density. 3D refinements for defocus were performed in M.

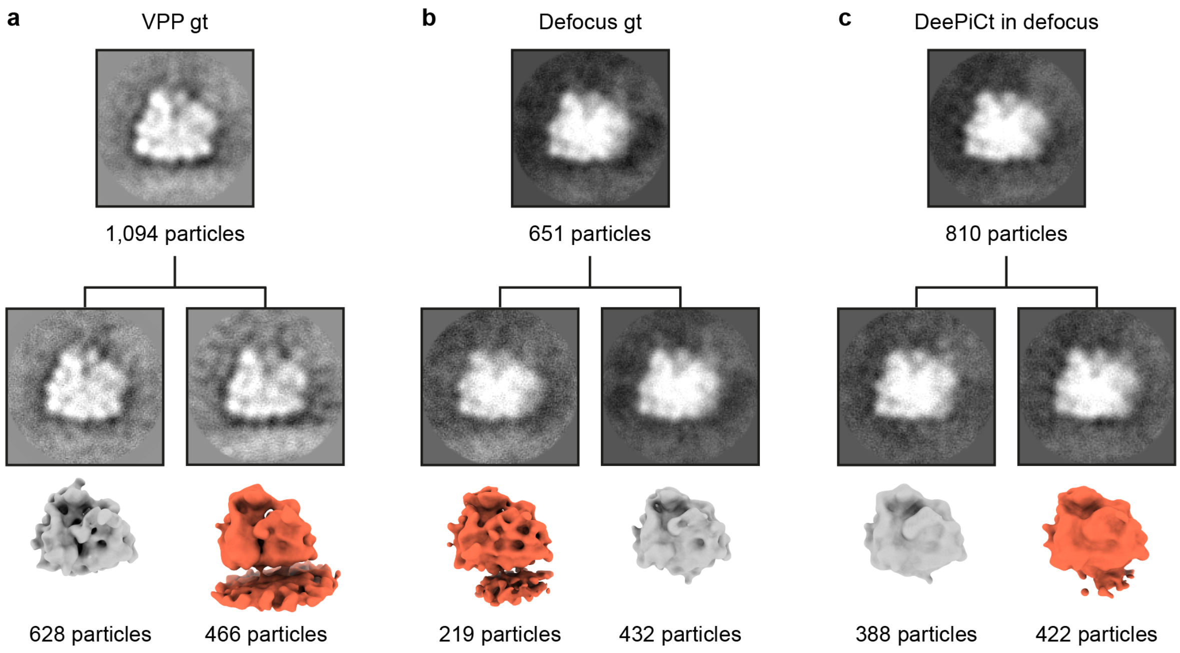

Supplementary Figure 11 | 3D classifications and refinements of ER-bound ribosomes in VPP and defocus ground truth, and DeePiCt predictions in defocus datasets. a-c. 2D slices through 3D-refined subtomogram average of ribosomes in 25 nm distance to the ER recovered from VPP ground truth (gt), defocus gt or DeePiCt predictions on defocus datasets. Focused 3D classifications (cyan sphere mask displayed in **Supplementary Fig. 10a**) separated in all 3 datasets one class (orange) with membrane density. 3D refinements for defocus were performed in M.

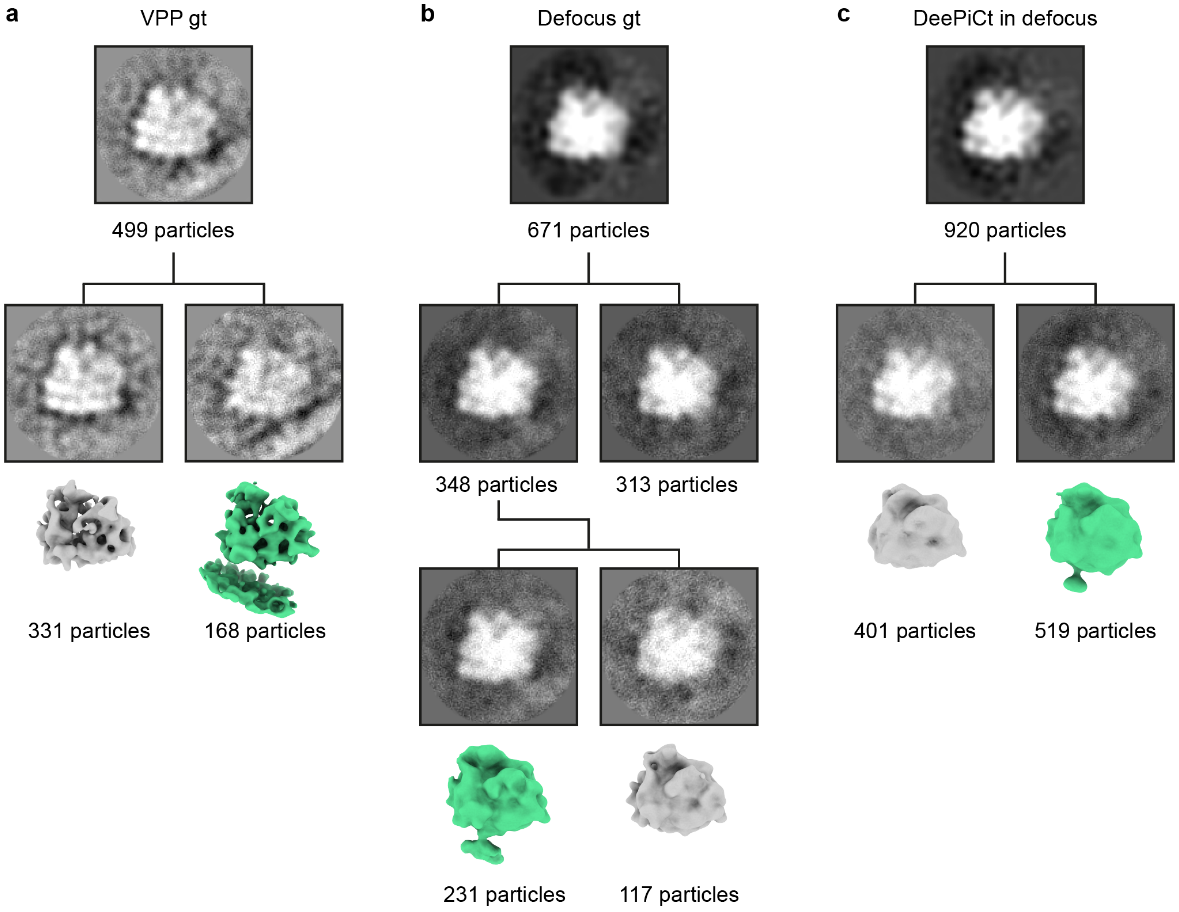

Supplementary Figure 12 | 3D classifications and refinements of mitochondria-bound ribosomes in VPP and defocus ground truth, and DeePiCt predictions in defocus datasets. a-c. 2D slices through 3D-refined subtomogram average of ribosomes in 25 nm distance to the mitochondria recovered from VPP ground truth (gt), defocus gt or DeePiCt prediction on defocus datasets. Focused 3D classifications (cyan sphere mask displayed in **Supplementary Fig. 10a**) separated in all 3 datasets one class (green) with membrane density. 3D refinements for defocus were performed in M.

Supplementary Table 7 | Cryo-EM data collection for VPP tomograms, refinement and statistics for ground truth subtomogram averages

	#1 FAS	#2 Ribosome all	#3 Ribosome LSU	#4 Ribosome with 40S head density	#5 Ribosome with exit tunel density	#6 Ribosome close to ER	#7 Ribosome close to mitochon dria	#8 Ribosome exclusively detected by DeePiCt
	(EMD-14404)	(EMD-14408)	(EMD-14409)	(EMD-14410)	(EMD-14411)	(EMD-14406)	(EMD-14405)	-
Magnificatio n	42,000	42,000	42,000	42,000	42,000	42,000	42,000	42,000
Voltage (kV)	300	300	300	300	300	300	300	300
Electron exposure (e–/Å^2)	113.6	113.6	113.6	113.6	113.6	113.6	117.1	113.6
Defocus range (μm)	-2.0 to -4.0	-2.0 to -4.0	-2.0 to -4.0	-2.0 to -4.0	-2.0 to -4.0	-2.0 to -4.0	-2.5 to -4.0	-2.0 to -4.0
Pixel size (Å)	3.3702	3.3702	3.3702	3.3702	3.3702	3.3702	3.3702	3.3702
Symmetry imposed	D3	C1	C1	C1	C1	C1	C1	C1
Initial particle images (no.)	731	25,311	25,311	25,311	25,311	1,094	499	5,554
Final particle images (no.)	731	25,311	4,812	3,482	5,755	466	168	5,554
Map resolution (Å) FSC threshold 0.143	26	16	25	21	20	31	31	22

Supplementary Table 8 | Cryo-EM data collection for defocus tomograms, refinement and statistics for ground truth subtomogram averages

	#1 FAS	#2 Ribosome all	#3 Ribosome well-aligned	#4 Ribosome with 40S head density	#5 Ribosome with exit tunel density	#6 Ribosome close to ER	#7 Ribosome close to mitochondria	#8 Ribosome exclusively detected by DeePiCt
	(EMD-14412)	(EMD-14417)	(EMD-14419)	(EMD-14419)	(EMD-14420)	(EMD-14415)	(EMD-14413)	-
Magnification	42,000	42,000	42,000	42,000	42,000	42,000	42,000	42,000
Voltage (kV)	300	300	300	300	300	300	300	300
Electron exposure (e–/Å^2)	120.3	120.3	120.3	120.3	120.3	120.3	120.3	120.3
Defocus range (μm)	-2.0 to -4.0	-2.0 to -4.0	-2.0 to -4.0	-2.0 to -4.0	-2.0 to -4.0	-2.0 to -4.0	-3.5 to -4.0	-2.0 to -4.0
Pixel size (Å)	3.3702	3.3702	3.3702	3.3702	3.3702	3.3702	3.3702	3.3702
Symmetry imposed	D3	C1	C1	C1	C1	C1	C1	C1
Initial particle images (no.)	366	25,901	25,901	25,901	25,901	651	671	4,283
Final particle images (no.)	366	25,901	10,970	3,601	3,349	219	231	4,283
Map resolution (Å) FSC threshold 0.143	27	11	9.3	12	11	31	31	15

Supplementary Table 9 | Cryo-EM data collection for defocus tomograms, refinement and statistics for subtomogram averages based on DeePiCt predictions

	#1 FAS	#2 Ribosome all	#3 Ribosome well-aligned	#4 Ribosome with 40S head density	#5 Ribosome with exit tunel density	#6 Ribosome close to ER	#7 Ribosome close to mitochondria
	(EMD-14422)	(EMD-14425)	(EMD-14426)	-	-	(EMD-14424)	(EMD-14423)
Magnification	42,000	42,000	42,000	42,000	42,000	42,000	42,000
Voltage (kV)	300	300	300	300	300	300	300
Electron exposure (e–/Å^2)	120.3	120.3	120.3	120.3	120.3	120.3	120.3
Defocus range (μm)	-2.0 to -4.0	-2.0 to -4.0	-2.0 to -4.0	-2.0 to -4.0	-2.0 to -4.0	-2.0 to -4.0	-3.5 to -4.0
Pixel size (Å)	3.3702	3.3702	3.3702	3.3702	3.3702	3.3702	3.3702
Symmetry imposed	D3	C1	C1	C1	C1	C1	C1
Initial particle images (no.)	108	26,866	26,866	26,866	26,866	810	920
Final particle images (no.)	108	26,866	5,815	3,348	1,503	422	519
Map resolution (Å) FSC threshold 0.143	28	15	9.4	15	16	29	34

Bibliography

70. Zeiler, M. D., Krishnan, D., Taylor, G. W. & Fergus, R. Deconvolutional networks. in *2010 IEEE Computer Society Conference on Computer Vision and Pattern Recognition* 2528–2535 (IEEE, 2010). doi:10.1109/CVPR.2010.5539957.

71. Milletari, F., Navab, N. & Ahmadi, S.-A. V-Net: Fully Convolutional Neural Networks for Volumetric Medical Image Segmentation. in *2016 Fourth International Conference on 3D Vision (3DV)* 565–571 (IEEE, 2016). doi:10.1109/3DV.2016.79.

72. Sudre, C. H., Li, W., Vercauteren, T., Ourselin, S. & Jorge Cardoso, M. Generalised dice overlap as a deep learning loss function for highly unbalanced segmentations. in *Deep learning in medical image analysis and multimodal learning for clinical decision support* (eds. Cardoso, M. J. et al.) vol. 10553 240–248 (Springer International Publishing, 2017).

73. Consortium, M. MONAI: Medical Open Network for AI. *Zenodo* (2022) doi:10.5281/zenodo.6903385.

74. Kingma, D. P. & Ba, J. Adam: A Method for Stochastic Optimization. https://arxiv.org/abs/1412.6980 (2017).

75. van der Walt, S. *et al.* scikit-image: image processing in Python. *PeerJ* **2**, e453 (2014).

76. Danev, R., Buijsse, B., Khoshouei, M., Plitzko, J. M. & Baumeister, W. Volta potential phase plate for in-focus phase contrast transmission electron microscopy. *Proc Natl Acad Sci USA* **111**, 15635–15640 (2014).

77. Buijsse, B., Trompenaars, P., Altin, V., Danev, R. & Glaeser, R. M. Spectral DQE of the Volta phase plate. *Ultramicroscopy* **218**, 113079 (2020).
