# Supplementary material for: Convolutional networks for supervised mining of molecular patterns within cellular context
Source: Nat Methods. 2023 Jan 23;20(2):284–94. doi: 10.1038/s41592-022-01746-2 (PMC9911354; doi:10.1038/s41592-022-01746-2)
Supplement: Supplementary file 2 — Reporting Summary [file 41592_2022_1746_MOESM2_ESM.pdf]

nature research

Corresponding author(s): Judith Zaugg, Julia Mahamid

Last updated by author(s): Nov 21, 2022

Reporting Summary

Nature Research wishes to improve the reproducibility of the work that we publish. This form provides structure for consistency and transparency in reporting. For further information on Nature Research policies, see our Editorial Policies and the Editorial Policy Checklist.

Statistics

For all statistical analyses, confirm that the following items are present in the figure legend, table legend, main text, or Methods section.

n/a	Confirmed	
☐	☒	The exact sample size (*n*) for each experimental group/condition, given as a discrete number and unit of measurement
☐	☒	A statement on whether measurements were taken from distinct samples or whether the same sample was measured repeatedly
☐	☒	The statistical test(s) used AND whether they are one- or two-sided<br>*Only common tests should be described solely by name; describe more complex techniques in the Methods section.*
☐	☒	A description of all covariates tested
☒	☐	A description of any assumptions or corrections, such as tests of normality and adjustment for multiple comparisons
☐	☒	A full description of the statistical parameters including central tendency (e.g. means) or other basic estimates (e.g. regression coefficient) AND variation (e.g. standard deviation) or associated estimates of uncertainty (e.g. confidence intervals)
☐	☒	For null hypothesis testing, the test statistic (e.g. *F*, *t*, *r*) with confidence intervals, effect sizes, degrees of freedom and *P* value noted<br>*Give P values as exact values whenever suitable.*
☒	☐	For Bayesian analysis, information on the choice of priors and Markov chain Monte Carlo settings
☒	☐	For hierarchical and complex designs, identification of the appropriate level for tests and full reporting of outcomes
☒	☐	Estimates of effect sizes (e.g. Cohen's *d*, Pearson's *r*), indicating how they were calculated

Our web collection on statistics for biologists contains articles on many of the points above.

Software and code

Policy information about availability of computer code

Data collection	SerialEM 3.7 or 3.8: Mastronarde, D.N. 2005. Automated electron microscope tomography using robust prediction of specimen movements. J. Struct. Biol. 152:36-51 (doi:10.1016/j.jsb.2005.07.007)
Data analysis	The DeePiCt code is assembled as two Snakemake pipelines (2D CNN and 3D CNN); the 3D CNN is implemented in the Pytorch framework while the 2D CNN is implemented in Keras, both in Python 3. The code, trained models, and link to the Google Colab notebook are available in the github repository https://github.com/ZauggGroup/DeePiCt. Cryo-electron tomograms were reconstructed with IMOD (version 4.9.4, doi: 10.1006/jsbi.1996.0013). Structural data analysis was performed with Warp (version 1.0.9, doi: 10.1038/s41592-019-0580-y), M (version 1.0.9, doi: 10.1038/s41592-020-01054-7), Relion (version 3.0.7, doi:10.7554/eLife.42166), Dynamo (version 1.1.520, doi: 10.1107/S2059798317003369), and TOM toolbox release-2008 (doi: 10.1016/j.jsb.2004.10.006) implemented in MATLAB 2016b & 2019b (https://www.mathworks.com). Subtomogram averages were visualized with UCSF Chimera (version 1.16.0, doi: 10.1002/jcc.20084) and UCSF ChimeraX (version 1.4.0, doi: 10.1002/pro.3235).

For manuscripts utilizing custom algorithms or software that are central to the research but not yet described in published literature, software must be made available to editors and reviewers. We strongly encourage code deposition in a community repository (e.g. GitHub). See the Nature Research guidelines for submitting code & software for further information.

Data

Policy information about availability of data

All manuscripts must include a data availability statement. This statement should provide the following information, where applicable:

- Accession codes, unique identifiers, or web links for publicly available datasets
- A list of figures that have associated raw data
- A description of any restrictions on data availability

Raw tilt series, tomograms, ground truth coordinates and segmentations are available via EMPIAR accession codes EMPIAR-10988 (S. pombe) and EMPIAR-10989, EMD-16136 (RPE1). Subtomogram averages for S. pombe VPP and defocus ground truth annotations are available on EMDB (VPP ground truth: EMD-14404, EMD-14405, EMD-14406, EMD-14408, EMD-14409, EMD-14410, EMD-14411; defocus ground truth: EMD-14412, EMD-14413, EMD-14415, EMD-14417, EMD-14418, EMD-14419, EMD-14420; defocus DeePiCt predicted: EMD-14422, EMD-14423, EMD-14424, EMD-14425, EMD-14426).

Structural comparisons were performed with S. cerevisiae FAS (PDB: 2uv8), P. pastoris FAS (EMD-12139), eEF3 from S. cerevisiae (EMD-12062), the S. cerevisiae ribosome (EMD-1667) with the rRNA expansion segment ES27L (PDB-3izd), the nuclear export factor Arx1 bound to the 60S large ribosomal subunit S. cerevisiae (EMD-2169), the human Ebp1 (EMD-10608), S. cerevisiae ribosomes derived from extracted ER (EMD-3764), and the ER-bound HeLa ribosomes (EMD-8056). The large subunit (LSU, 60S) of a published S. cerevisiae 80S ribosome map (EMD-3228) and the S. cerevisiae FAS map (EMD-1623) were used as references for template matching. The HeLa cell dataset is available via accession code EMD-11992.

Field-specific reporting

Please select the one below that is the best fit for your research. If you are not sure, read the appropriate sections before making your selection.

☒ Life sciences ☐ Behavioural & social sciences ☐ Ecological, evolutionary & environmental sciences

For a reference copy of the document with all sections, see nature.com/documents/nr-reporting-summary-flat.pdf

Life sciences study design

All studies must disclose on these points even when the disclosure is negative.

Sample size	No sample size calculation was performed. From one sample of S. pombe cell culture, usually 4 cryo-grids were prepared. On each grid, around 5 lamellae with around 5 cells each could be prepared by cryo-FIB milling. On one lamella, up to 10 cryo-electron tomograms were collected. Thus, for cryo-ET sample preparation and data collection, the sample size for each experiment is sufficient. We chose 10 tomograms for training and testing. Our performance analysis showed that even less tomograms (depending on the particle to be predicted) are sufficient. Predicted particle numbers were sufficient for structural analysis, and in the case of ribosomes large enough to perform subsequent classifications.
Data exclusions	During sample preparation, only grid squares with several cells in the center, continuous support and sufficient ice thickness were chosen for lamella preparation. For cryo-electron tomography acquisition, areas without obstacles (e. g. crystalline ice) that are thin enough and therefore resulted in good image contrast and signal to noise ratio, were selected. Only high-quality tomograms were chosen based on thickness and residual error during tilt series alignment.
Replication	During sample preparation, usually 4 grids were prepared from which the ones most suitable for cryo-FIB milling were selected. The DeePiCt predictions were replicated in a robust cross-validation setting.
Randomization	For sample preparation, grids were prepared with cells from one cell culture condition and cryo-FIB milling performed in a non-targeted manner. For cryo-ET data collection, the selection of grids/lamellae/cells for final tomogram collection was not completely random because they were usually first selected for optimal cryo-FIB milling and areas on lamellae chosen based on ice thickness, etc. For regions meeting these quality standards that are required for high-quality cryo-ET data, the further selection process was randomized, without considerations of cytosolic content or other visible features. During structure refinement in M or RELION, particles were randomly divided into two half datasets by the software. For classification, at the particles were randomly divided evenly into the initial classes by RELION. For DeePiCt performance evaluation, the tomograms were used in cross-validation, no randomization needed.
Blinding	Researchers were not blinded during data collection to enable recording of cytosolic volumes which were already randomized by non-targeted cryo-FIB lamella creation. Researchers were not blinded for the analysis, and performance was evaluated by standard quality measures that were independent of subjective judgment.

Reporting for specific materials, systems and methods

We require information from authors about some types of materials, experimental systems and methods used in many studies. Here, indicate whether each material, system or method listed is relevant to your study. If you are not sure if a list item applies to your research, read the appropriate section before selecting a response.

Materials & experimental systems

n/a	Involved in the study
☒	☐ Antibodies
☒	☐ Eukaryotic cell lines
☒	☐ Palaeontology and archaeology
☒	☐ Animals and other organisms
☒	☐ Human research participants
☒	☐ Clinical data
☒	☐ Dual use research of concern

Methods

n/a	Involved in the study
☒	☐ ChIP-seq
☒	☐ Flow cytometry
☒	☐ MRI-based neuroimaging
